# Amyloid-PET and White Matter Hyperintensities Have Independent Effects on Baseline Cognitive Function and Synergistic Effects on Longitudinal Executive Function

**DOI:** 10.3390/brainsci13020218

**Published:** 2023-01-28

**Authors:** Doaa G. Ali, Erin L. Abner, Ahmed A. Bahrani, Riham El Khouli, Brian T. Gold, Yang Jiang, Donna M. Wilcock, Gregory A. Jicha

**Affiliations:** 1 Sanders-Brown Center on Aging, College of Medicine, University of Kentucky, Lexington, KY 40506, USA; 2 Department of Behavioral Science, College of Medicine, University of Kentucky, Lexington, KY 40506, USA; 3 Department of Epidemiology, College of Public Health, University of Kentucky, Lexington, KY 40536, USA; 4 Department of Neurology, College of Medicine, University of Kentucky, Lexington, KY 40506, USA; 5 Department of Radiology, College of Medicine, University of Kentucky, Lexington, KY 40536, USA; 6 Department of Neuroscience, College of Medicine, University of Kentucky, Lexington, KY 40536, USA; 7 Department of Physiology, College of Medicine, University of Kentucky, Lexington, KY 40506, USA

**Keywords:** white matter hyperintensities (WMH), regional standardized uptake value ratio (SUVr), executive function, neuroimaging, preclinical Alzheimer’s disease, cognitive function, neuroimaging

## Abstract

Co-occurrence of beta amyloid (Aβ) and white matter hyperintensities (WMHs) increase the risk of dementia and both are considered biomarkers of preclinical dementia. Moderation and mediation modeling were used to define the interplay between global and regional Aβ and WMHs measures in relation to executive function (EF) and memory composite scores outcomes at baseline and after approximately 2 years across a sample of 714 clinically normal participants from the Alzheimer’s Disease Neuroimaging Initiative (ADNI 2). The moderation regression analysis showed additive effects of Aβ and WMHs over baseline memory and EF scores (*p* = 0.401 and 0.061, respectively) and synergistic effects over follow-up EF (*p* < 0.05). Through mediation analysis, the data presented demonstrate that WMHs effects, mediated by global and regional amyloid burden, are responsible for baseline cognitive performance deficits in memory and EF. These findings suggest that Aβ and WMHs contribute to baseline cognition independently while WMHs volumes exert effects on baseline cognitive performance directly and through influences on Aβ accumulation.

## 1. Introduction

Alzheimer’s disease (AD) and subcortical vascular dementia are considered the most common pathologic contributors to dementia in the aging population. Both frequently coexist in over 80% of community dwelling adults with dementia [1]. Cerebral small vessel disease (CSVD) has also been linked to the pathogenesis of AD and is largely responsible for the development of subcortical vascular dementia [2]. AD and CSVD share multiple risk factors [3], occurring concomitantly in over 50% of individuals with dementia [4], and there may be substantial overlap between these two conditions in terms of clinical, pathological and radiological findings. Co-occurrence of beta amyloid (Aβ) (a hallmark pathologic feature of AD) and white matter hyperintensities (WMHs) (reflecting CSVD burden) increase the risk of dementia [5].

WMHs and Aβ are also key drivers of cognitive decline in healthy older adults, and both are considered biomarkers of preclinical dementia [6,7]. Neuroimaging studies examining the combined impact of Aβ burden and WMHs on cognition have largely examined these variables independently rather than examining the potential interplay between these key pathologic hallmarks of dementia. Several studies have addressed this issue of interplay between Aβ and WMHs with often contradictory results [6,7,8,9,10,11,12,13,14]. Notably, many of these studies focused on global measures of WMHs and Aβ without exploration of regional effects in relation to stage of disease. Studying the effect of Aβ and WMHs on cognition without emphasizing the importance of lesion location can miss the functional consequences of the two pathologies. Previous studies investigating the impact of regional distribution of WMHs on executive function (EF) found that WMHs in all cortical regions (frontal areas, occipital, parietal and temporal) are associated with deficits in EF scores [15,16,17], but regional quantification of Aβ and its interaction with WMH volume has not been fully investigated in these prior studies.

Aβ deposition is hypothesized as being the initial step in the neuropathological development of AD and dementia [18], but findings from previous studies have also shown that WMHs often occur prior to the presence of amyloid-β plaques in preclinical AD [19,20,21] supporting a retrograde degeneration hypothesis [22,23]. Additionally, evidence from recent studies has suggested that the relationship between WMHs and Aβ is strongly determined by the spatial distribution of the two pathologies [24]. The majority of published studies have emphasized the spatial heterogeneity of WMHs [25,26], but have left the potential heterogeneous influences of regional Aβ deposition relatively unexplored.

Since mixed dementia is widely recognized as the norm rather than the exception, in the present study we sought to explore the relation between regional and global Aβ and WMHs with cognitive function (EF and memory) scores in cognitively normal (CN) older adults at baseline, and further examine the relation between WMHs and regional Aβ deposition in relation to cognitive performance changes over time.

## 2. Materials and Methods

Data used in the preparation of this article were obtained from the Alzheimer’s Disease Neuroimaging Initiative (ADNI) database (adni.loni.usc.edu). The ADNI was launched in 2003 as a public–private partnership, led by Principal Investigator Michael W. Weiner, MD. The primary goal of ADNI has been to test whether serial magnetic resonance imaging (MRI), positron emission tomography (PET), other biological markers and clinical and neuropsychological assessment can be combined to measure the progression of mild cognitive impairment (MCI) and early Alzheimer’s disease (AD).

### 2.1. Participants

Summary data from the Alzheimer’s Disease Neuroimaging Initiative (ADNI 2) at http://adni.loni.usc.edu (accessed on 24 July 2021) [27] were used in the present analyses. Only participants with a mini-mental state examination (MMSE) score greater than or equal to 26 at baseline meeting these inclusion criteria were included in the analysis: (1) complete demographic information (i.e., age, sex, education) and ApoE genotype available; (2) neurocognitive composite metrics for EF and memory at baseline and after approximately 2 years, (3) PET Florbetapir for focal and global Aβ quantification; and (4) T2 FLAIR scan acquisition with WMHs volume quantification. Details of ADNI inclusion criteria, clinical procedures and methodology are available elsewhere [28,29]. These criteria were developed to specifically investigate predisease and preclinical disease states that may lead to further cognitive impairment and dementia.

Participants with missing data required for the analyses were excluded. A total of 53 participants were excluded from the longitudinal analysis on the basis of missing data required for the analysis. Excluded participants did not differ significantly from these included in the analysis in regards to age, sex, education, ApoE or baseline MMSE scores (data not shown).

### 2.2. Imaging Analysis

#### 2.2.1. White Matter Hyperintensity Quantification Method

WMH volumes were quantified using the 4-tissue segmentation method described previously [30]. Briefly, first step was co-registration of the FLAIR to the 3D T1 image inhomogeneity-corrected and non-linearly aligned to a minimal deformation template (MDT) using the T1 transformation and the FMRIB Software Library (FSL) toolbox [31,32]. Modified Bayesian probability and structure prior probability maps were used to estimate WMH in the MDT. Binary WMHs masks were then created using 3.5 SD threshold above the mean. The segmented WMHs masks were then back-transformed into native space for tissue WMH volumes calculation. An Expectation–Maximization (EM) algorithm was used for segmentation to isolate gray, white, and CSF measurements in template-space. Transforming these masks back to each image’s native space produced rough estimate 3-tissue segmentations. Finally, WMHs were ultimately subtracted from segmented white matter volume and reported in cubic millimeters.

#### 2.2.2. Calculation of Florbetapir Cortical Summary Values

Preprocessing of the AV-45 PET scans and computation of the global AV-45 PET values were done centrally by the ADNI core as described previously [33]. Briefly, each subject’s florbetapir image was coregistered using SPM8 to that subject’s MRI image that was closest in time to the florbetapir scan. Freesurfer processing was carried out to skull-strip, segment and delineate cortical and subcortical regions in all MRI scans [34,35]. Volume-weighted florbetapir means from a cortical summary region were extracted. A single binary cortical summary region composed of all the subregions was created to calculate the mean uptake across each region. We used the summary data of global and regional results (frontal, parietal and cingulate regions that have been most frequently associated with EF performance [36,37,38,39]). SUVr determination was based on the whole cerebellum reference region. Details regarding regions of interest forming the subregions frontal, parietal and cingulate have been described previously [33].

### 2.3. Composite Measures of Executive Function and Memory: ADNI_EF and ADNI_Memory

The specific tests included in ADNI executive function (ADNI-EF) composite scores are Category Fluency (animal and vegetable naming), Trail Making Tests A and B, Digit Span backwards, Wechsler adult intelligence scale-revised (WAIS-R) Digit Symbol Substitution and 5 Clock Drawing items (circle, symbol, numbers, hands, time) [40]. The memory composite (ADNI_Memory) included the Rey auditory verbal learning test (RAVLT), the cognitive component of the Alzheimer’s disease assessment scale (ADAS-Cog) and Wechsler logical memory scale scores [40]. We used the ADNI-EF and ADNI-Memory scores corresponding to the baseline scan and EF and memory scores within one to three years from the baseline as follow-up scores.

### 2.4. Statistical Analyses

All statistical analyses were conducted using SPSS, version 26.0 (SPSS Inc., Chicago, IL, USA). Significance was set at *p* < 0.05, with correction based on the number of the analysis. We used multiple regression models including the interaction term and the main effects of Aβ and WMHs to investigate the independent and combined associations of these pathologies with cognition (ADNI-EF and ADNI-Memory). WMH volumes were logarithmically transformed due to the positive skewed distribution for the statistical analysis. Moderation analyses assessing the relationship between Aβ and WMHs were fit to the data using the following equation:

Ŷ = b_0_ + b_1_WMHs + b_2_ Aβ + b_3_WMH*Aβ +**bX**

where Ŷ represents the mean scores of composite measures of cognitive (executive or memory) function and **bX** represents beta coefficients and adjustment covariates. Age (continuous values), sex (indicator variable), education (continuous values), ApoE (dummy indicator variable) and total intracranial volumes (continuous values) were entered as covariates in all analyses. The models were built using Hayes’ PROCESS macro for SPSS (model 1) [41]. Longitudinal analyses examined the association of the interaction between baseline Aβ burden and WMHs with mean follow-up cognitive (ADNI-EF or ADNI-Memory) scores after controlling for baseline ADNI cognitive scores and time following the baseline scan acquisitions and cognitive testing. To consider the regional Aβ burden effects in addition to global Aβ burden and WMHs on baseline and change in ADNI-EF and ADNI-Memory, moderated regressions were run for amyloid deposition (SUVr) in each of the a priori selected cortical regions (Frontal, cingulate and parietal), adjusting for age, sex, education, ApoE and intracranial volumes.

Assuming that WMH is causally related to Aβ deposition, which is in turn causally related to cognitive (ADNI-EF or ADNI-Memory) scores, mediation models were built to estimate the relationship between WMH (independent variable) and cognitive function (dependent variable) and Aβ (mediator variable). This model was built using Hayes’ PROCESS macro for SPSS (model 4) [41]. Significance was tested using 5000 bootstrap samples to calculate bias-corrected 95% confidence intervals. Indirect effects with bootstrapped 95% confidence intervals not crossing zero were considered significant. Age, sex, education, ApoE and intracranial volumes were entered as covariates in the mediation regression models. Baseline ADNI composite scores and times between the two cognitive scores tests were added as covariates in the mediation regression models examining longitudinal cognitive score changes.

## 3. Results

The demographic, clinical and imaging characteristics of the included participants are provided in Table 1. Briefly, the sample included 326 women and 388 men with a mean age of 73.13 ± 7.4 years; participants were highly educated on average (16.3 ± 2.6 years).

### 3.1. Testing the Interaction Term and the Main Effect of Global and Regional Aβ Burden and WMHs with Cognition

**Figure 1:**
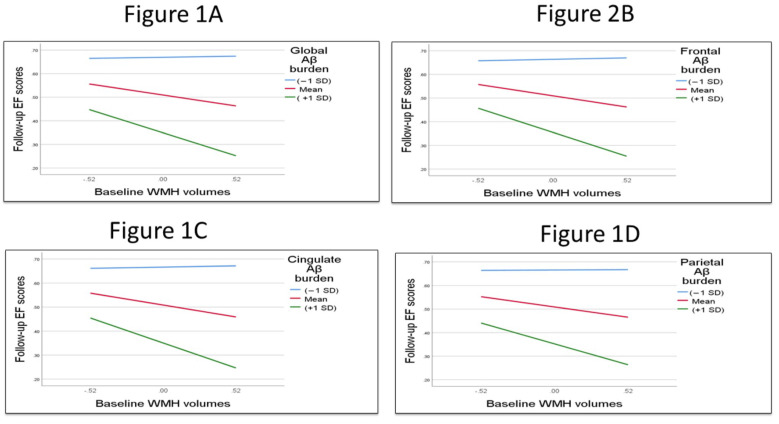
Longitudinal EF performance at low (−1 SD), moderate mean (0 SD) and high (1 SD) levels of baseline global and regional Aβ burden and WMHs.

The moderation regression analysis detected a marginally statistically significant interaction effect between Aβ burden and WMHs in relation to baseline EF (*p*-value = 0.061), and a significant interaction term between the biomarkers in relation to longitudinal executive functions with *p*-value = 0.036. Overall, when amyloid burden was higher, the association between log WMHs and ADNI-EF was stronger. (+1 SD above sample mean) (Figure 1A). Regarding memory composite scores, the models did not detect any statistically significant interaction effect between global Aβ burden and WMHs in relation to baseline ADNI-memory scores or follow-up ADNI-memory scores (*p*-Value 0.401 and 0.937 respectively).

The regional models examining the interaction between Aβ burden in discrete cortical regions and WMH volumes detected marginally significant interaction between frontal, parietal and cingulate Aβ burden and WMHs in relation to baseline EF performance with *p*-value 0.061, 0.059 and 0.071, respectively. In contrast, the models failed to detect even marginally significant interaction effects between regional Aβ burden and WMHs with baseline memory (*p*-values for interaction term were in frontal Aβ 0.360, parietal Aβ 0.353 and cingulate Aβ 0.411).

The moderation analysis detected a significant interaction effect of frontal and cingulate Aβ burden and WMHs on longitudinal EF performance with *p* value 0.033 for frontal and 0.024 for cingulate (Figure 1B,C). The (regression) slopes illustrating the negative association of WMH volume with longitudinal executive function performance for three levels of regional Aβ burden and WMHs (−1, 0 and 1 SD using sample’s mean) are shown in Figure 1. In contrast to the results for EF, the moderation regression analysis failed to detect a statistically significant interaction effect between regional Aβ burden and WMH in relation to follow-up ADNI-memory scores. Results regarding the moderation models between global and regional Aβ burden and WMHs with baseline and follow-up cognitive (ADNI-EF and ADNI-Mem) scores are displayed in Supplementary tables.

The (regression) slopes representing WMH’s negative associations with executive functions for high levels of regional Aβ burden (+1 SD using sample’s mean). Figure 1A showed the interaction term of global Aβ burden and WMHs with Follow-up ADNI-EF scores. Overall, when amyloid burden was higher, the association between log WMH and ADNI-EF was stronger. (+1SD above sample mean). Figure 1B showed the interaction term of frontal Aβ burden and WMHs with Follow-up ADNI-EF scores. Figure 1C showed the interaction term of baseline cingulate Aβ burden and WMHs with Follow-up ADNI-EF scores. Figure 1D showed the interaction term of baseline parietal Aβ burden and WMHs with Follow-up ADNI-EF scores.

### 3.2. Testing the Mediation Effects of Global and Regional Aβ Burden on WMH-Related Cognitive Performance

**Figure 2:**
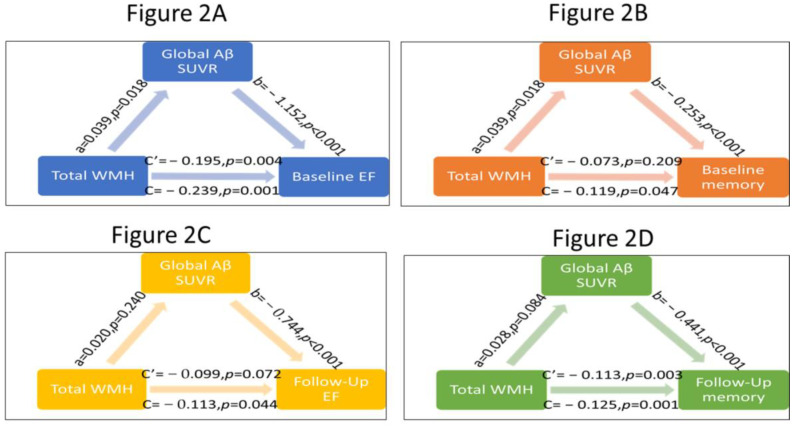
Path models of the mediation effect of global and Aβ burden between WMHs and cognitive performance.

Mediation analyses were conducted to assess whether global and regional Aβ burdens act as potential mediators of the relationship between WMHs and cognitive performance. Path models of the mediation effect are presented in Table 2. The path analysis revealed that global Aβ deposition mediates the relationship between WMHs and baseline cognitive (EF and memory) scores (see Figure 2A,B). The bootstrap confidence interval (CI) for the indirect effect demonstrated that the indirect effect of WMH on baseline EF performance (a × b = −0.045; CI: −0.09, −0.01) and baseline memory scores (CI: −0.01, −0.006) through global Aβ burden were significant. Both frontal and parietal Aβ SUVr mediated the effect of WMH on baseline EF and memory performance. In contrast, there was no significant mediation effect by cingulate Aβ SUVr for baseline cognitive performance (see Table 2 and Supplementary Materials figures). The other model explored whether global and regional Aβ SUVr mediated the relationship between WMHs and follow-up cognitive (EF and memory) scores after adding baseline cognitive scores and times since the initial cognitive test as the covariates. Results demonstrated that neither global Aβ SUVr (see Figure 2C,D) nor regional Aβ SUVr mediates these associations as the CI for the indirect effect was crossing the zero and did not reach statistical significance. While our path analyses were conducted to assess whether global and regional Aβ burden act as potential mediators of the relationship between WMHs and ADNI-memory scores, we tested its opposite direction, and no significant mediating effects were detected for baseline or follow-up ADNI-memory scores. (See Supplementary Materials figures)

As such, path (a) expresses one unit increase in WMH associated with increase of Aβ SUVr, whereas path (b) expresses the change in cognitive function (EF and memory) associated with an increment of Aβ SUVr. Path (c’) shows the direct effect of WMHs on cognitive function scores and path (c) shows the total effect. The bootstrap confidence interval (CI) for the indirect effect demonstrated that the indirect effect of WMH on baseline EF performance (a × b = −0.045; CI: −0.09, −0.01) (Figure 2A) and baseline memory scores (CI: −0.01, −0.006) (Figure 2B) through global Aβ burden were significant. In contrast, the CI for the indirect mediation effects of WMHs on follow-up EF scores ((a × b = −0.015; CI: −0.045, 0.009) (Figure 2C)) and follow-up ADNI-memory scores ((CI: −0.031, 0.002) (Figure 2D)) through global Aβ SUVr were crossing zero. (WMH, white matter hyperintensities; SUVr, standard uptake value ratio; Aβ, beta amyloid; CI, confidence intervals).

### 3.3. Testing the Relation between WMHs and Global and Regional Aβ SUVr

**Figure 3:**
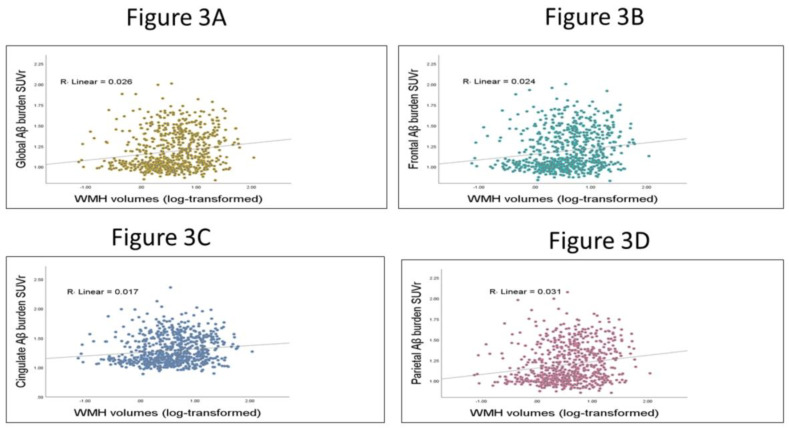
Scatterplot between global and regional Aβ burden and WMH volumes.

Bivariate plots were used to show the association between regional and global Aβ burden, and WMH volumes. (See Figure 3)

Figure 3: The correlation between regional and global Aβ burden, and WMH volumes.

The scatterplots show the relation between Aβ burden and WMH volumes. In Figure 3A, the Pearson correlation between global Aβ and WMH volumes was 0.162. Figure 3B, the Pearson correlation between frontal Aβ and WMH volumes was 0.155. In Figure 3C, the Pearson correlation between cingulate Aβ and WMH volumes was 0.130. Finally, in Figure 3D the Pearson correlation between parietal Aβ and WMH volumes was 0.177.

## 4. Discussion

The present results indicate that there is an independent effect of both Aβ and WMHs on cognitive performance at baseline and a synergistic interaction between these baseline biomarkers of pathology on future EF performance. This synergistic effect was not seen in future memory performance. The other main finding, demonstrated through mediation analysis, is that the extent of WMH-dependent changes in baseline EF was mediated by both global and regional Aβ burdens. Thus, WMH accumulation appears to increase Aβ deposition which in turn influences cognition longitudinally.

The present moderation analysis provides evidence of an additive effect of WMHs and Aβ in relation to baseline cognitive performance, which is consistent with the findings from other cross-section biomarker studies [6,9,10,42]. The present study extends the results from previous studies by analyzing the effect of baseline biomarkers of Aβ and WMHs on future cognitive performance. The data demonstrate that the combined effect of Aβ burden and WMHs on follow-up EF scores is greater than the sum of the two individual biomarker effects supporting a hypothesis of synergistic interactions between both Aβ and WMHs. The synergistic effect between the baseline biomarkers of pathology on follow-up EF scores was not seen in follow-up memory scores. This finding may be related to the population studied as ADNI participants are largely well-educated and ADNI excludes participants with severe CSVD. One previous study showed that CN participants with low WMH volumes underwent low to no changes in memory performance, independent of amyloid burden [11]. Additionally, findings from another study have demonstrated that the association between WMH volumes and both EF and memory function were significant in demented participants, but that the relationship between memory and WMHs was not detected in CN participants, such as those included in the present study [43]. It is also possible that the synergistic effect seen in follow-up EF performance but not memory performance may involve alterations in brain function and structure networks [44], WMH burden and/or Aβ deposition that were not included in the present analysis.

The present data further demonstrate that regional Aβ burden in frontal and cingulate regions are most related to WMHs effect on future EF change. These data suggest that this relation might be explained by WMHs’ impacts on connectivity within the executive control, and default mode networks [44]. Indeed, the present findings support the hypothesis that executive function performance is dependent on both intact white matter pathway connectivity, as well as pathologic integrity of the cortical regions themselves [12,45,46] and that there is a synergistic interaction between baseline biomarkers of pathology on longitudinal cognitive functions that is dependent on frontal–subcortical circuitry in cognitive aging and pAD [13].

Studying the independent associations of WMHs and Aβ burden with baseline and follow-up cognitive performance, the present data demonstrate that baseline EF performance is explained by both global amyloid burden and WMHs, but that Aβ burden was the only predictor of follow-up EF performance and baseline memory performance (See Supplementary Tables S1 and S2). Consistent with our findings, one recent study from the University of California, Davis Alzheimer’s Disease Research Center (UCD ADRC) diversity cohort found that WMHs were related to baseline EF, but not longitudinal change in EF [11]. Conversely, the same group has argued that baseline WMHs likely represent only a small percentage of the final WMHs [47], suggesting that longitudinal EF performance may be most related to progression of WMHs, rather than solely associated with baseline WMHs. It is also possible that these findings are related to the population studied, as ADNI excludes participants with severe vascular risk factors that may limit analysis of the full impact of WMH and other cerebrovascular injury in this cohort [9,48]. It should also be pointed out that baseline WMH was one of the predictors for follow-up memory performance with Aβ burden. These findings emphasize the important role of WMHs as a risk factor for future memory decline even if they may not affect baseline memory performance at the stage of pAD [11,49].

The present data clearly demonstrate that WMHs are associated with global and regional Aβ burden pathology, exerting an effect on baseline cognitive performance through multiple pathways. Consistent with our findings, a recently published study based on the C6 project in the Medical Imaging Trial Network of Canada (MITNEC-C6), which differs from ADNI in being a WMH-enriched cohort [50] demonstrated that the relation between global WMH and cognition was mediated by global Aβ burden and cortical atrophy. The authors interpreted their results to suggest that Aβ burden appears to be aggravated in participants with WMHs supporting a synergistic pathophysiological process. They further hypothesized that the relationship between WMHs and Aβ burden may be related to impaired general cerebral perfusion [5,51] or to vascular pathology affecting amyloid clearance pathways [8]. Another possible explanation presented by the authors focused on myelin damage [52], which has been shown to promote Aβ oligomerization, eventually contributing to amyloid deposition [53]. These potential mechanisms may help explain the topographic patterns of regional Aβ deposition in relation to WMH effects on critical brain networks seen in the present study. The hypothesis of a regional association between Aβ and WMHs is supported by the present data.

One major limitation of the present study includes the possibility of selection bias as ADNI excludes participants with significant evidence for cerebrovascular disease, which may limit discoveries into the interactions between Aβ and WMHs that may exist in more generalizable samples. Further studies examining the interaction between Aβ and WMHs in participants with higher levels of CSVD including lacunes, small subcortical infarctions, microinfarcts and microbleeds are needed to more fully investigate the associations of Aβ with CSVD. Additionally, the sample of participants evaluated is relatively small for moderation analyses but was restricted by the availability of extant data. Longitudinal outcome analyses may also be limited by the relatively short follow-up period in the ADNI 2 participants studied. It is possible that a more extended follow-up period would help to clarify further the relationships between Aβ and WMHs across progressive stages of cognitive decline, including MCI and early dementia stages. Strengths of the present study include the use of ADNI data allowing analysis in a large sample of relatively homogeneous CN participants that have been classified extensively for the presence of pAD and CSVD. An additional strength is the use of both mediation and moderation analyses in studying the relation between cognitive function and the effects of the two primary drivers of dementia in the population (Aβ and WMHs) within discrete brain regions.

## 5. Conclusions

While to what extent WMHs affect Aβ burden and cognition remain unclear, the present data demonstrate that baseline Aβ burden and WMH volumes have both independent associations with EF and memory scores at baseline, as well as synergistic associations with future EF performance. The extent of WMH-dependent changes in baseline cognitive performance was related to both the direct effect of WMHs and an indirect effect moderated through global and regional Aβ burdens. The biological relationships between regional Aβ and WMHs responsible for this indirect effect need further investigation across a broader profile of the disease course of dementia.

## Figures and Tables

**Table 1:** Characteristics of participants.

Characteristic	Range	Mean (SD)	Sample Size
Age (years)	55–96	73.13 (7.41)	714
Education (years)	10–20	16.31 (2.6)	714
Global Aβ burden (SUVr)	0.84–2.01	1.19 (0.22)	714
Frontal Aβ burden (SUVr)	0.83–2.01	1.19 (0.23)	714
Cingulate Aβ burden (SUVr)	0.89–2.36	1.29 (0.22)	714
Parietal Aβ burden (SUVr)	0.87–2.08	1.20 (0.22)	714
WMH volumes in cubic centimeters	0.07–61.02	6.94 (8.54)	714
WMH volumes (log-transformed)	−1.13 to 1.79	0.57 (0.52)	714
Total intracranial volumes	1084.29 to 1861.82	411.88 (135.97)	714
ADNI-EF (baseline)	−3.01 to 2.99	0.54 (0.94)	714
ADNI-EF (follow-up)	−3.01 to 2.99	0.50 (1.06)	661
ADNI-memory (baseline)	−2.8 to 3.14	0.56 (0.81)	714
ADNI-memory (follow-up)	−2.62 to 3.06	0.52 (0.94)	661
Time between test (years)	1 to 3	1.86 (0.49)	661
APOE e4 allele N(%) copy ^1^	2 to 4	58 (8.1)	714
APOE e4 allele N(%) copy ^2^	3 to 4	300 (42.1)	714
Sex male N (%)		388 (54.3)	714

Key: Aβ, Beta Amyloid; WMH, White Matter Hyperintensity; ADNI-EF, Executive Function; SD, Standard Deviation; SUVr, Standardized Uptake Value Ratio.

**Table 2:** The mediation effects of global and regional Aβ burden on WMH-related cognitive performance.

		Path (a) Coefficient β (*p*-Value)	Path (b) Coefficient β (*p*-Value)	Direct Effect of WMH Path (c’) Coefficient β (*p*-Value)	Total Effect Path (c) Coefficient β (*p*-Value)	CI for Indirect Effect
Cognitive Function	Aβ Burden					
Baseline EF	Global Aβ SUVr	0.039 (0.018)	−1.152 (<0.001)	−0.195 (0.004)	−0.239 (0.001)	−**0.09 and** −**0.01**
	Frontal Aβ SUVr	0.037 (0.032)	−1.093 (<0.001)	−0.199 (0.003)	−0.239 (0.001)	−**0.008 and** −**0.005**
	Cingulate Aβ SUVr	0.031 (0.085)	−0.979 (<0.001)	−0.216 (0.002)	−0.239 (0.001)	−0.057 and 0.02
	Parietal Aβ SUVr	0.052 (0.007)	−1.114 (<0.001)	−0.182 (0.007)	−0.239 (0.001)	−**0.11 and** −**0.02**
Baseline memory	Global Aβ SUVr	0.039 (0.018)	−1.253 (<0.001)	−0.073 (0.209)	−0.119 (0.047)	−**0.01 and** −**0.006**
	Frontal Aβ SUVr	0.037 (0.033)	−1.148 (<0.001)	−0.077 (0.178)	−0.119 (0.047)	−**0.11 and** −**0.01**
	Cingulate Aβ SUVr	0.03 (0.085)	−1.013 (0.001)	−0.089 (0.121)	−0.119 (0.047)	−0.07 and 0.004
	Parietal Aβ SUVr	0.052 (0.002)	−1.174 (<0.001)	−0.059 (0.302)	−0.119 (0.047)	−**0.11 and** −**0.02**
Follow-up EF scores	Global Aβ SUVr	0.02 (0.24)	−0.744 (<0.001)	−0.099 (0.072)	−0.113 (0.044)	−0.045 and 0.009
	Frontal Aβ SUVr	0.018 (0.314)	−0.689 (<0.001)	−0.102 (0.061)	−0.113 (0.044)	−0.045 and 0.009
	Cingulate Aβ SUVr	0.016 (0.385)	−0.685 (<0.001)	−0.101 (0.065)	−0.113 (0.044)	−0.04 and 0.014
	Parietal Aβ SUVr	0.029 (0.088)	−0.717 (<0.001)	−0.092 (0.093)	−0.113 (0.044)	−0.05 and 0.01
Follow-up memory scores	Global Aβ SUVr	0.028 (0.084)	−0.441 (<0.001)	−0.113 (0.003)	−0.125 (0.001)	−0.031 and 0.002
	Frontal Aβ SUVr	0.026 (0.127)	−0.418 (<0.001)	−0.114 (0.004)	−0.125 (0.001)	−0.028 and 0.04
	Cingulate Aβ SUVr	0.021 (0.238)	−0.373 (<0.001)	−0.117 (0.002)	−0.125 (0.001)	−0.024 and 0.05
	Parietal Aβ SUVr	0.039 (0.019)	−0.408 (<0.001)	−0.109 (0.004)	−0.125 (0.001)	−0.035 and 0.002

Path (a) expresses the change in Aβ SUVr with one unit increase in WMH, whereas path (b) expressed decrement of cognitive function associated with an increment of Aβ SUVr. Path (c’) shows the direct effect of WMH on cognitive function and path (c) shows the total effect. Indirect effects with bootstrapped 95% confidence intervals not crossing zero were considered significant (bold in the table).

## Data Availability

Summary data from the Alzheimer’s Disease Neuroimaging Initiative (ADNI 2) could be found at http://adni.loni.usc.edu. Data accessed on 24 July 2021.

## Supplementary Materials

The following supporting information can be downloaded at https://www.mdpi.com/article/10.3390/brainsci13020218/s1, Figure S1: Path models of the mediation effect of WMHs between global and regional Aβ burden and baseline ADNI-memory scores; Figure S2: Path models of the mediation effect of baseline WMH volumes on follow-up ADNI-memory performance; Table S1: The interaction between global and regional Aβ burden and WMHs on cross-section and longitudinal performance in EF; Table S2: The interaction term and the main effect of global and regional Aβ burden and WMHs with memory score.
