# Supplementary material for: Amyloid-PET and White Matter Hyperintensities Have Independent Effects on Baseline Cognitive Function and Synergistic Effects on Longitudinal Executive Function
Source: Brain Sci. 2023 Jan 28;13(2):218. doi: 10.3390/brainsci13020218 (PMC9953773; doi:10.3390/brainsci13020218)
Supplement: Supplementary file 1 [file brainsci-13-00218-s001.zip › Supplementary figures.pdf]

Figure S1. Path models of the mediation effect of global and regional Aβ burden between WMH and baseline EF performance.

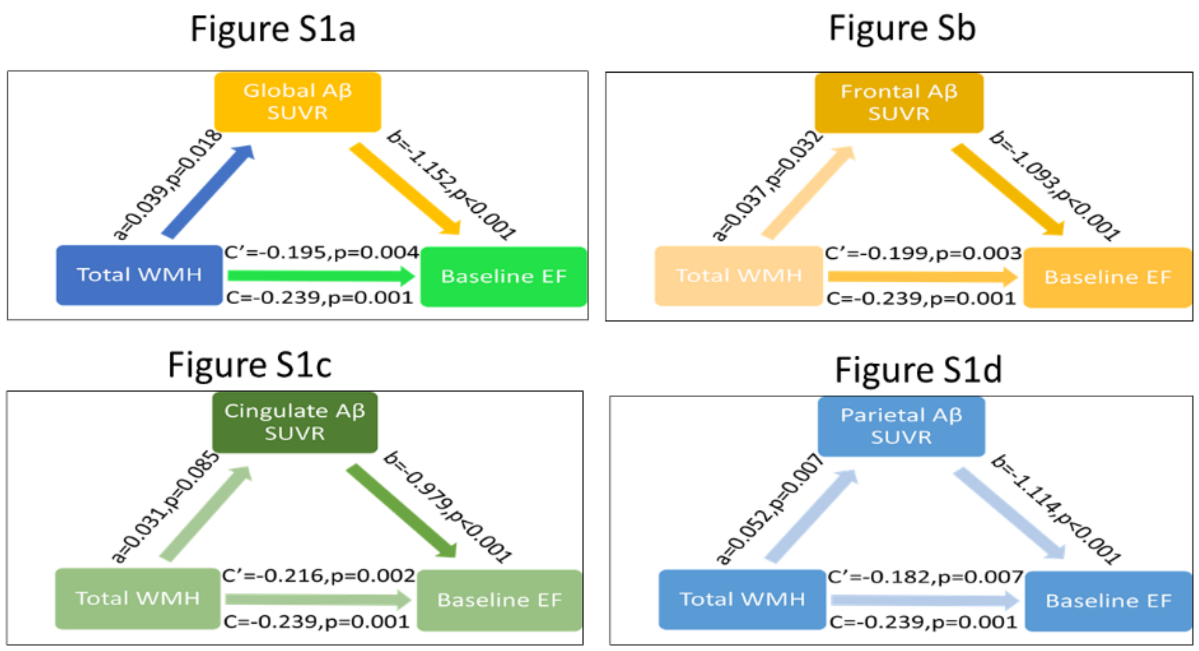

As such, path a is expressed the change in Aβ SUVr with one unit increase in WMH, whereas path b is expressed decrement of baseline EF associated with an increment of Aβ SUVr. Path c' the direct effect of WMH on EF and path c (total effect). The confidence intervals for the indirect mediation effects of WMH on baseline EF through global Aβ burden (a× b = −0.045; CI: −0.09, −0.01) (Figure S1a), frontal Aβ (a× b = −0.045; CI: −0.08, −0.005) (Figure S1b) and parietal Aβ (a× b = −0.058; −0.11, −0.02) (Figure S1d) were significant. There was no significant mediation effect by cingulate Aβ SUVr for baseline EF performance (Figure S1c).

(WMH, white matter hyperintensities; EF, Executive function; SUVr, standard uptake value ratio; Aβ, beta amyloid)

Figure S2: Path models of the mediation effect of global and regional Aβ burden between WMH and baseline ADNI-memory scores.

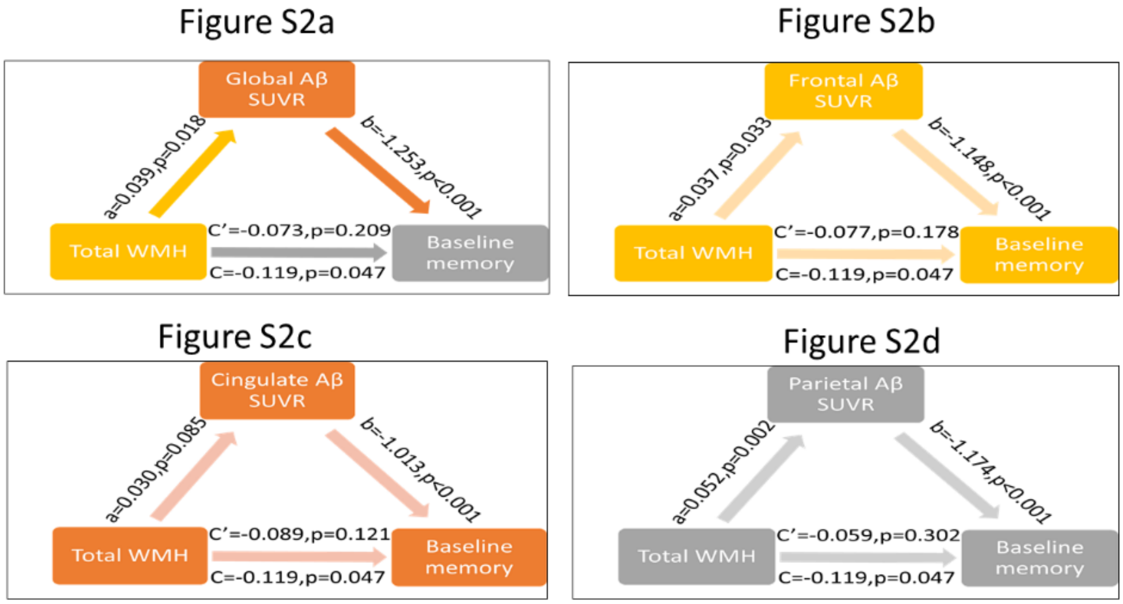

As such, path a is expressed the change in Aβ SUVr with one unit increase in WMH, whereas path b is expressed decrement of baseline ADNI-memory scores associated with an increment of Aβ SUVr. Path c' the direct effect of WMH on ADNI-memory scores and path c (total effect). The confidence intervals (CI) for the indirect mediation effects of WMH on baseline ADNI-memory scores through global (CI: −0.01, -0.006) (Figure S2a), frontal Aβ (CI: −0.11, -0.01) (Figure S2b) , cingulate Aβ (CI: −0.07, 0.004) (Figure S2c), and parietal Aβ (CI: −0.11, -0.02) (Figure S2d). (WMH, white matter hyperintensities; SUVr, standard uptake value ratio; Aβ, beta amyloid; CI, confidence intervals).

Figure S3. Path models of the mediation effect of baseline global and regional Aβ burden on longitudinal EF performance.

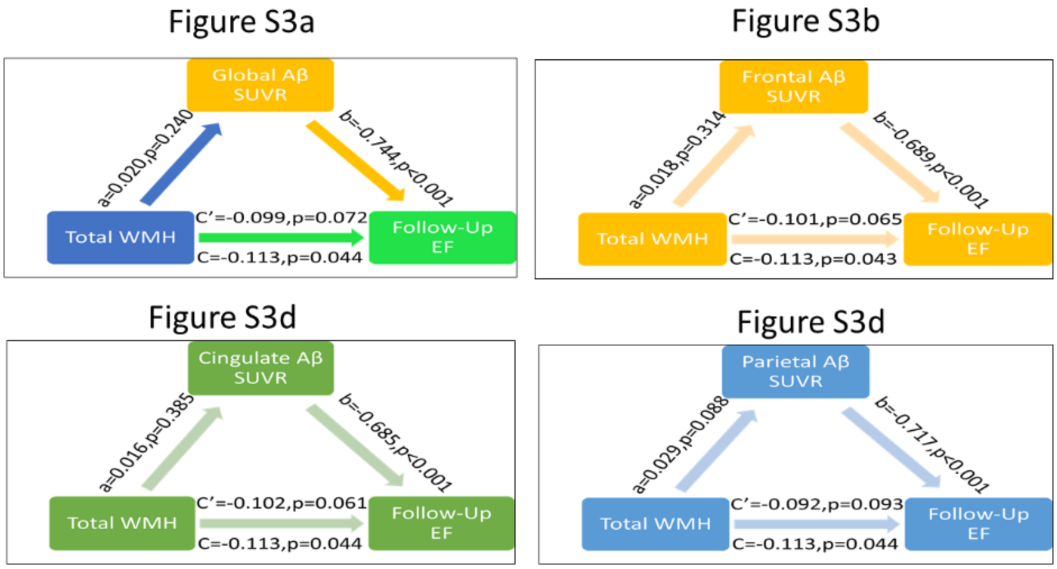

As such, path a is expressed one unit increase in WMH associated with increase of Aβ SUVr , whereas path b is expressed the change in longitudinal EF associated with an increment of Aβ SUVr. Path c' the direct effect of WMH on EF and path c (total effect). The confidence intervals (CI) for the indirect mediation effects of WMH on follow-up EF scores through global Aβ SUVr (($a\times b = -0.015$; CI :−0.045, 0.009) (Figure S3a), frontal Aβ ($a\times b = -0.012$; CI: −0.041, 0.011) (Figure S3b), cingulate Aβ ($a\times b = -0.011$; CI: −0.04, 0.014) (Figure S3c), and parietal Aβ ($a\times b = -0.021$; CI: −0.05, 0.01) (Figure S3d). (WMH, white matter hyperintensities; EF, Executive function; SUVr, standard uptake value ratio; Aβ, beta amyloid; CI, confidence intervals).

Figure S4. Path models of the mediation effect of baseline global and regional Aβ burden on longitudinal memory performance.

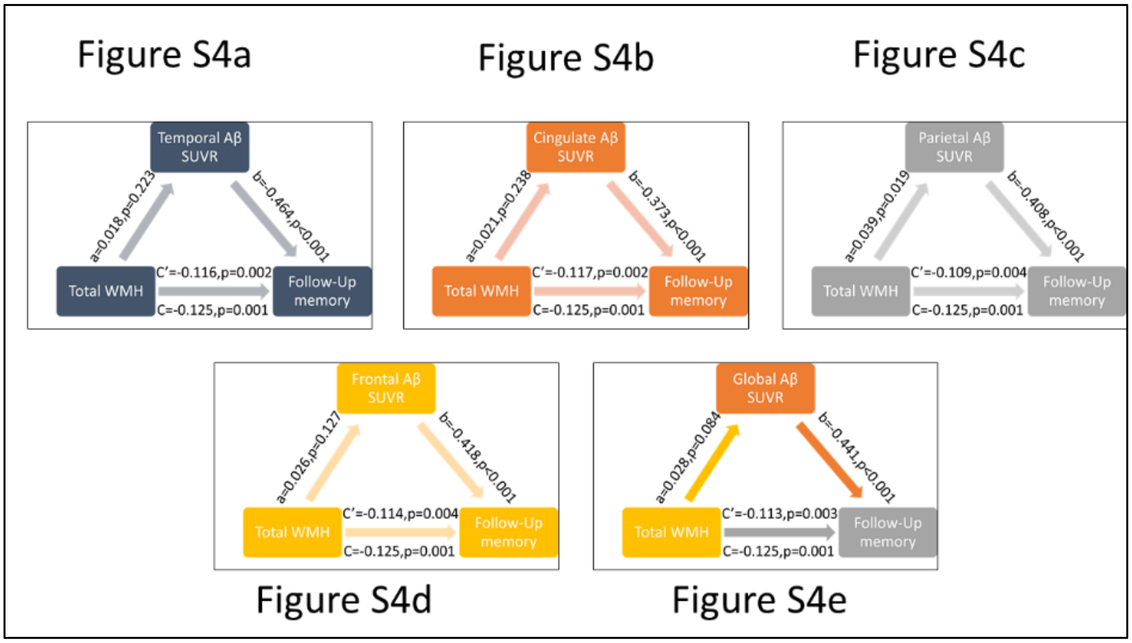

As such, path a is expressed one unit increase in WMH associated with increase of Aβ SUVr, whereas path b is expressed the change in follow-up ADNI-memory scores associated with an increment of Aβ SUVr. Path c' the direct effect of WMH on follow-up ADNI-memory scores and path c (total effect). The confidence intervals (CI) for the indirect mediation effects of WMH on follow-up ADNI-memory scores through temporal Aβ (CI:-0.026, 0.06) (Figure S4a), cingulate Aβ (CI: −0.024, 0.05) (Figure S4b), parietal Aβ (CI: −0.035, 0.002) (Figure S4c), frontal Aβ (CI: −0.028, 0.04) (Figure S4d), and global Aβ (CI: −0.031, 0.002) (Figure S4e) were crossing zero. (WMH, white matter hyperintensities; SUVr, standard uptake value ratio; Aβ, beta amyloid; CI, confidence intervals).

Supplementary figure S5: Path models of the mediation effect of WMH between global and regional Aβ burden and baseline ADNI-memory scores.

Figure S5a

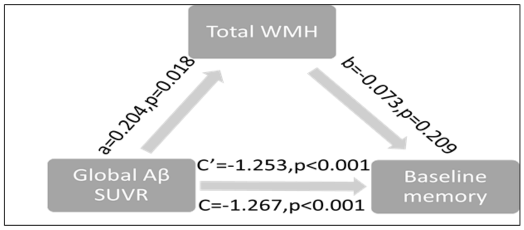

Figure S5b

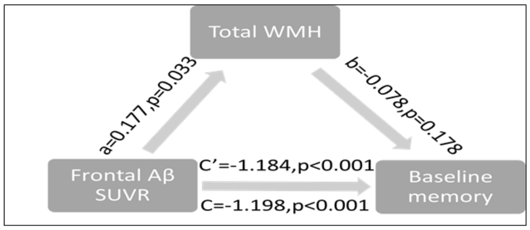

Figure S5c

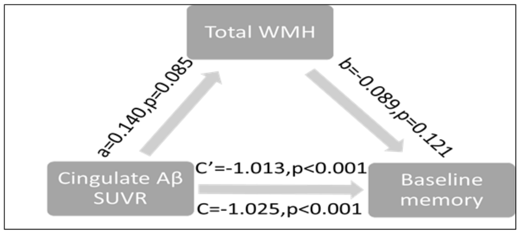

Figure S5d

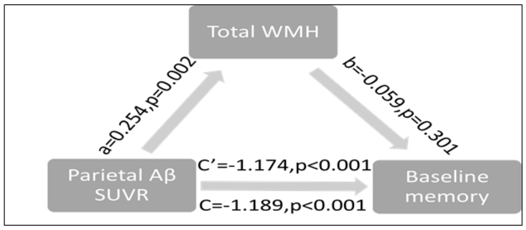

As such, path a is expressed the change in WMH volumes with one unit increase in Aβ SUVr, whereas path b is expressed decrement of baseline ADNI-memory scores associated with an increment of WMH. Path c’ the direct effect of Aβ SUVr on ADNI-memory scores and path c (total effect). The confidence intervals (CI) for the indirect mediation effects of WMH on baseline ADNI-memory scores through global (CI: −0.043, 0.007), frontal Aβ (CI: −0.039, 0.05), cingulate Aβ (CI: −0.039, 0.04), and parietal Aβ (CI: −0.046, 0.012) were crossing zero indicating no significant mediating effects.

Supplementary figure S6: Path models of the mediation effect of baseline WMH volumes on follow-up ADNI-memory performance

Figure S6a Figure S6b Figure S6c

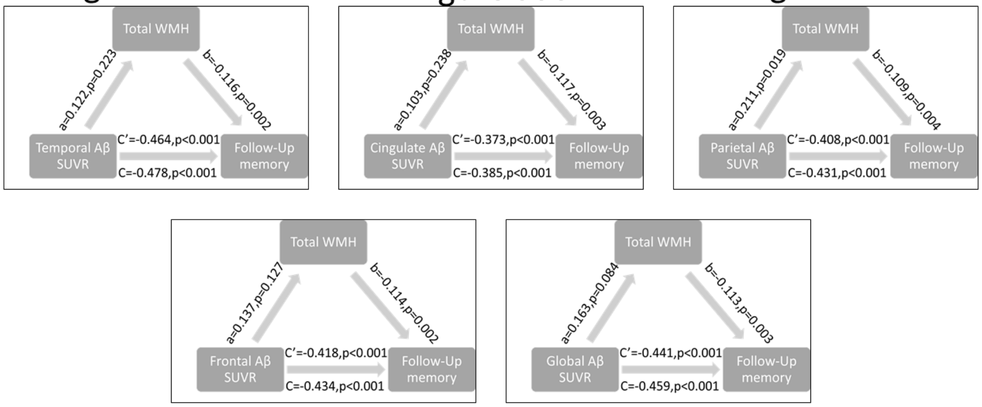

Figure S6d Figure S6e

As such, path (a) is expressed one unit increase in Aβ SUVr associated with increase of WMH volumes, whereas path (b) is expressed the change in follow-up ADNI-memory scores associated with an increase in WMH volumes. (Path c') the direct effect of Aβ SUVr on follow-up ADNI-memory scores and path c (total effect). The confidence intervals (CI) for the indirect mediation effects of WMH on follow-up ADNI-memory scores through temporal Aβ (CI:-0.04, 0.01) cingulate Aβ (CI: −0.036, 0.009), parietal Aβ (CI: −0.052, 0.0001), frontal Aβ (CI: −0.024, 0.006), and global Aβ (CI: −0.047, 0.04) were crossing zero indicating no significant mediating effects.
