# Supplementary material for: Amyloid-PET and White Matter Hyperintensities Have Independent Effects on Baseline Cognitive Function and Synergistic Effects on Longitudinal Executive Function
Source: Brain Sci. 2023 Jan 28;13(2):218. doi: 10.3390/brainsci13020218 (PMC9953773; doi:10.3390/brainsci13020218)
Supplement: Supplementary file 1 [file brainsci-13-00218-s001.zip › Supplementary tables.pdf]

Supplementary table S1: The interaction between global and regional Aβ burden and WMHs on cross-section and longitudinal performance in EF

	Baseline EF		Longitudinal EF	
	Coefficient	P value	Coefficient	P value
Main effect of WMH	-0.198	0.003*	-0.073	0.177
Main effect of Global SUVr	-1.162	<0.001**	-0.708	<0.001**
Interaction WMH * Global SUVr	0.517	0.061	-0.482	0.029*
Main effect of WMH	-0.203	0.003*	-0.094	0.097
Main effect of Frontal SUVr	-1.104	<0.001**	-0.672	<0.001**
Interaction between WMH * frontal SUVr	0.496	0.061	-0.452	0.033*
Main effect of WMH	-0.185	0.006*	-0.086	0.112
Main effect of Parietal SUVr	-1.126	<0.001**	-0.701	<0.001**
Interaction between WMH * parietal SUVr	0.505	0.059	-0382	0.052
Main effect of WMH	-0.217	0.015*	-0.098	0.144
Main effect of Cingulate SUVr	-0.801	<0.001**	-0.678	<0.001**
Interaction between WMH *cingulate SUVr	0.488	0.071	-0.455	0.024*

Key: WMH, white matter hyperintensities; EF, Executive function; SUVr, standardized uptake value ratio; ** less than 0.001, * less than 0.05

Supplementary table S2: The interaction term and the main effect of global and regional Aβ burden and WMHs with memory score:

	Baseline ADNI_Memory score		Follow-up ADNI_Memory score	
	Coefficient	P_ value	coefficient	P_ value
Main effect of WMH	-0.0753	0.202	-0.108	0.004
Main effect of Global SUVr	-1.257	<0.001**	-0.439	<0.001**
Interaction WMH * Global SUVr	0.196	0.401	-0.012	0.937
Main effect of WMH	-0.078	0.170	-0.109	0.003*
Main effect of Frontal SUVr	-1.189	<0.001**	-0.491	<0.001**
Interaction WMH * frontal SUVr	0.205	0.360	-0.051	0.917
Main effect of WMH	-0.063	0.291	-0.105	0.005*
Main effect of Parietal SUVr	-1.179	<0.0001**	-0.405	<0.001**
Interaction between WMH * parietal SUVr	0.209	0.353	0.021	0.887
Main effect of WMH	-0.079	0.119	-0.113	0.003*
Main effect of Cingulate SUVr	-1.015	<0.001**	-0.369	<0.001**
Interaction between WMH *cingulate SUVr	0.188	0.411	-0.007	0.923

Main effect of WMH	-0.083	0.14	-0.112	0.003*
Main effect of temporal SUVr	-1.366	<0.001**	-0.464	<0.001**
WMH*Temporal SUVr	0.208	0.406	-0.026	0.873

Key: WMH, white matter hyperintensities; EF, Executive function; SUVr, standardized uptake value ratio; ** less than 0.001, * less than 0.05
