# Supplementary figures and images for: Amyloid-PET and White Matter Hyperintensities Have Independent Effects on Baseline Cognitive Function and Synergistic Effects on Longitudinal Executive Function
Source: Brain Sci. 2023 Jan 28;13(2):218. doi: 10.3390/brainsci13020218 (PMC9953773; doi:10.3390/brainsci13020218)

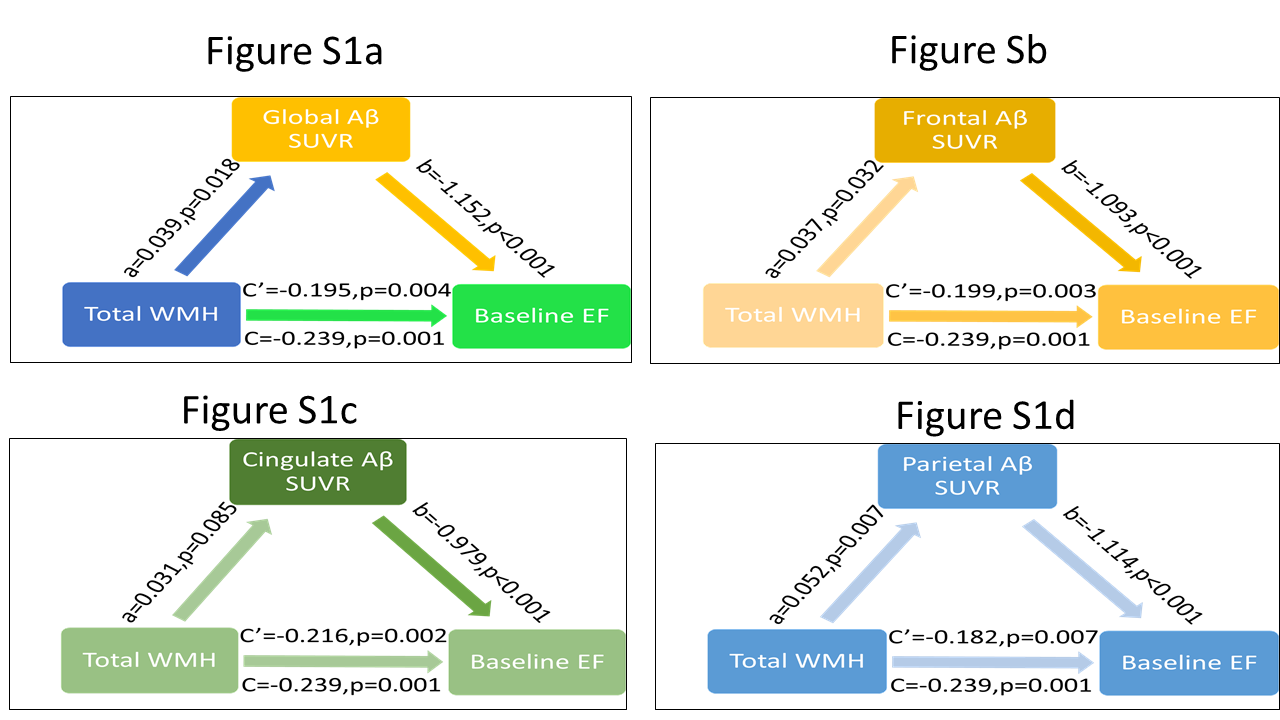

Supplement: Supplementary file 1 [file brainsci-13-00218-s001.zip › Figure s1300.tif]

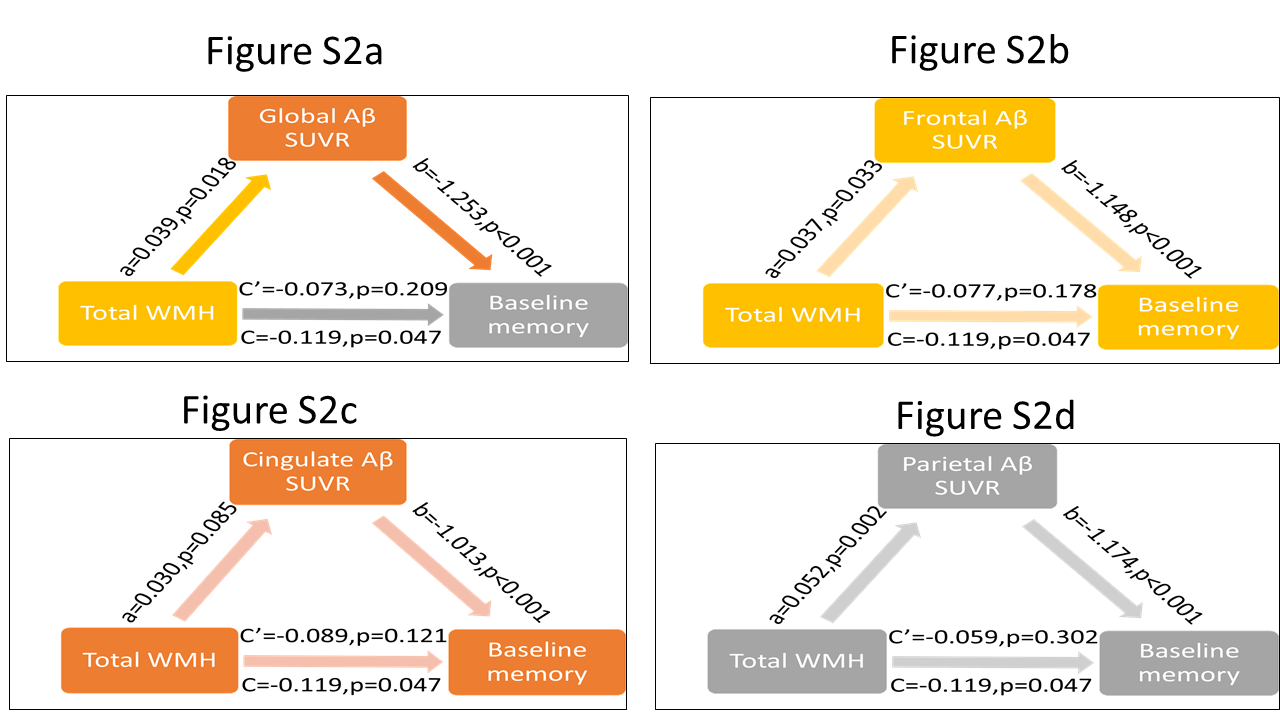

Supplement: Supplementary file 1 [file brainsci-13-00218-s001.zip › Figure S2300.tif]

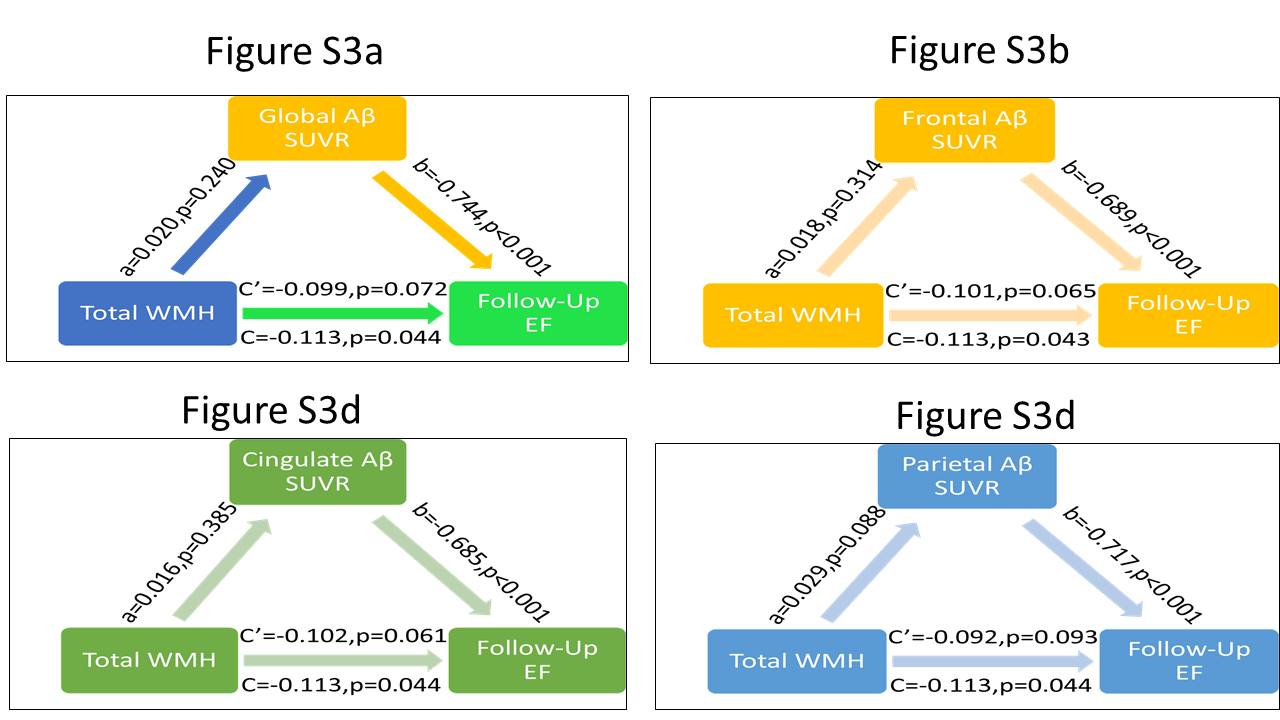

Supplement: Supplementary file 1 [file brainsci-13-00218-s001.zip › Figure S3300.tif]

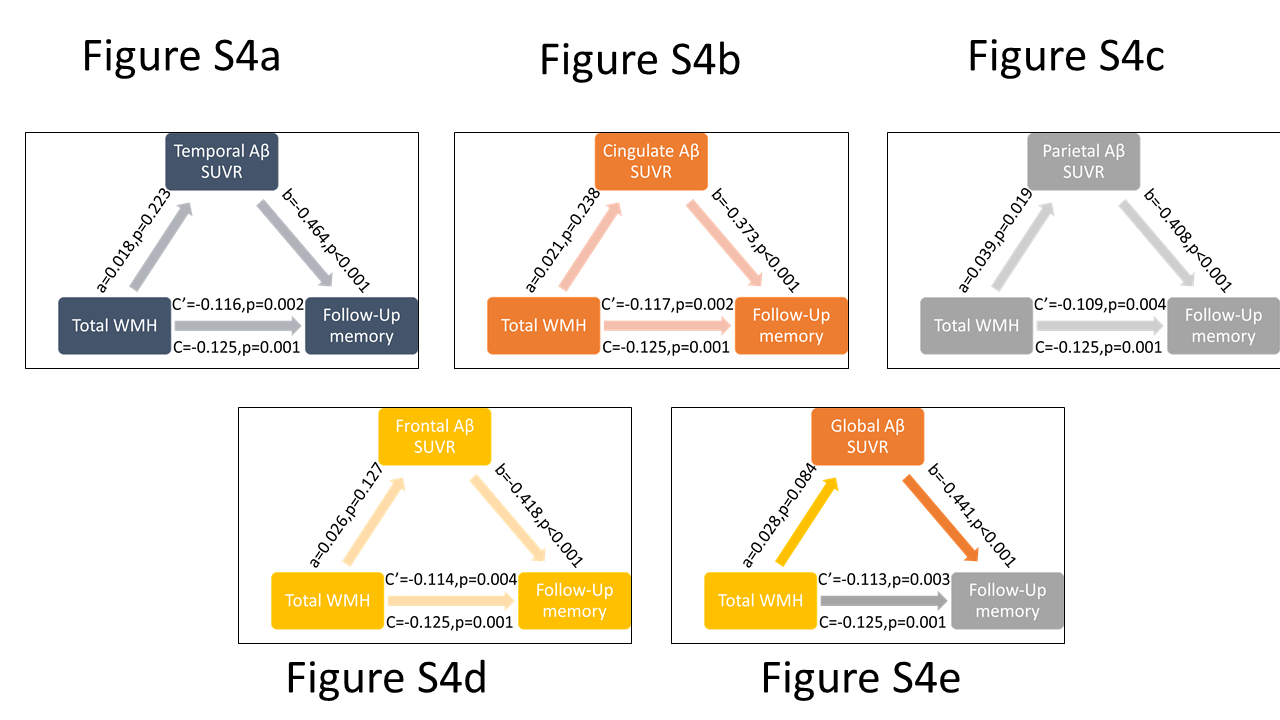

Supplement: Supplementary file 1 [file brainsci-13-00218-s001.zip › Figure S4300.tif]

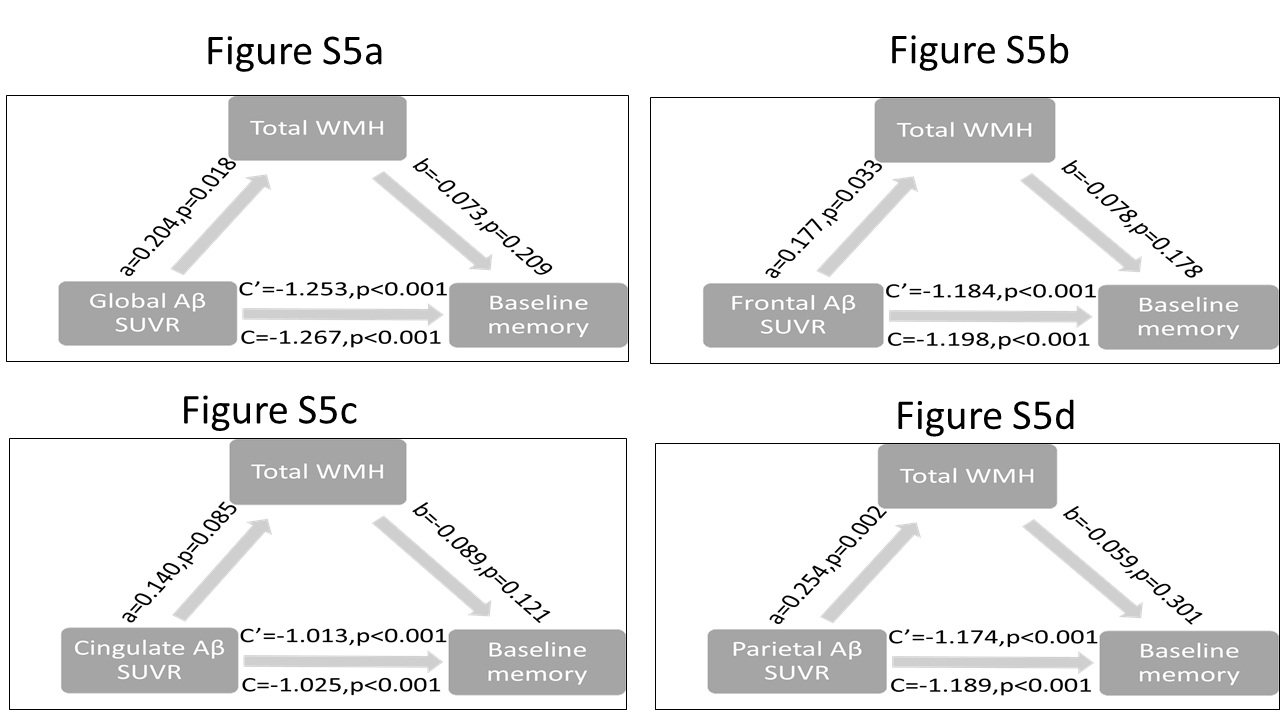

Supplement: Supplementary file 1 [file brainsci-13-00218-s001.zip › Figure S5300.tif]

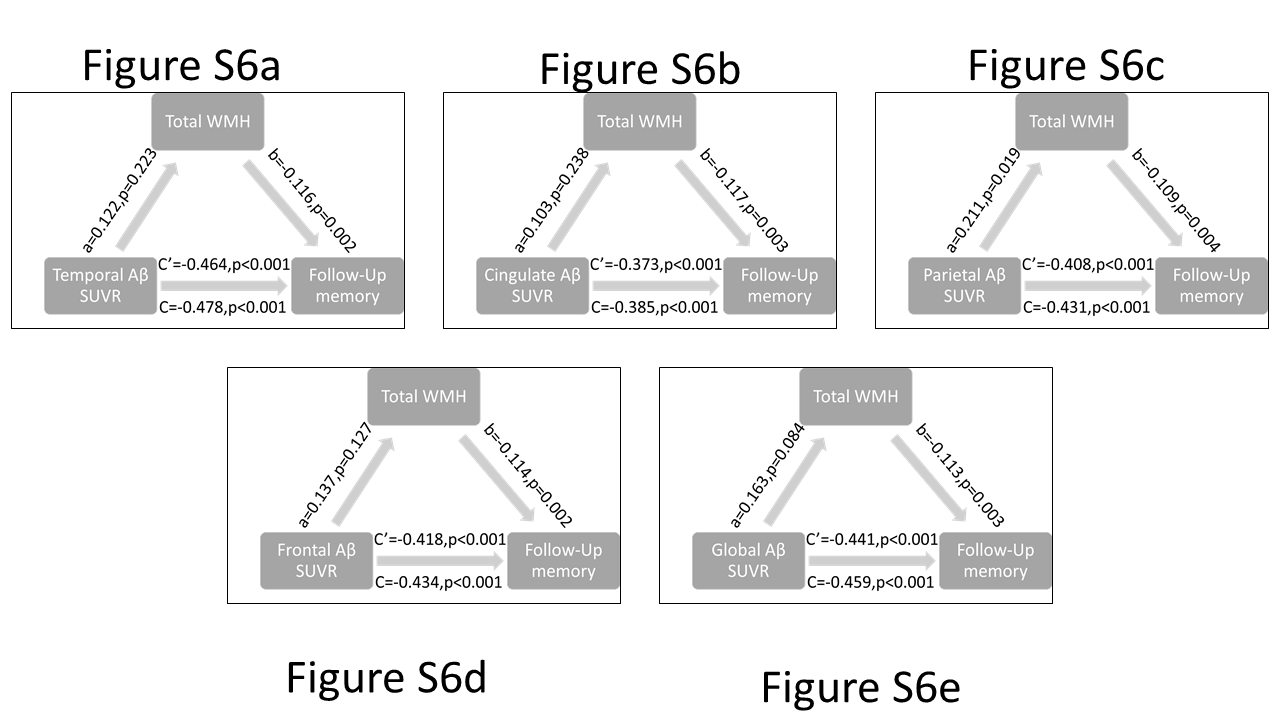

Supplement: Supplementary file 1 [file brainsci-13-00218-s001.zip › figure S6300.tif]
